# Confined spaces in space: Cerebral implications of chronic elevations of inspired carbon dioxide and implications for long‐duration space travel

**DOI:** 10.1113/EP091659

**Published:** 2025-01-07

**Authors:** Jay M. J. R. Carr, Philip N. Ainslie, Trevor Day

**Affiliations:** ^1^ Centre for Heart, Lung and Vascular Health University of British Columbia Okanagan Kelowna BC Canada; ^2^ Department of Biology Mount Royal University Calgary AB Canada

**Keywords:** cerebral blood flow, chronic hypercapnia, respiratory acidosis

## Abstract

Cerebrovascular regulation is critically dependent upon the arterial partial pressure of carbon dioxide ($P_{\mathrm{aC}O_{2}}$), owing to its effect on cerebral blood flow, tissue $P_{CO_{2}}$, tissue proton concentration, cerebral metabolism and cognitive and neuronal function. In normal environments and in the absence of pathology, at least over acute time frames, hypercapnia is usually managed readily via the respiratory chemoreflex arcs and/or acid–base buffering capacity, such that there is minimal impact on cerebrovascular and neurological function. However, in non‐normal environments, such as enclosed spaces, or with pathology, extended exposures to elevations in $P_{\mathrm{aC}O_{2}}$ can be detrimental to cerebral health. Given the direct effect of protons on cellular function, even if pH is normalized, it is feasible that higher proton concentrations could still produce detrimental effects. Although it seems that humans can work safely in mildly hypercapnic environments for extended periods, chronic respiratory acidosis can cause bone demineralization, renal calcification, perinatal developmental abnormalities, systemic inflammation and impairments in cognitive function and visuomotor skills and can produce cerebral acidosis, potentially inducing sustained alterations in cerebral function. With the advancement of new initiatives in spaceflight, including proposed long‐duration missions to Mars, the study of the effects of chronic inspired CO_2_ on human health is relevant. In this review, we draw on evidence from preclinical, physiological and clinical research in humans to summarize the cerebral ramifications of prolonged and chronic exposures to elevated partial pressures of inspired CO_2_ and respiratory acidosis.

## Abstract (highlights)

- **What is the topic of this review?**
  What are the known impacts of chronic hypercapnia on integrated cardiorespiratory and cerebrovascular function and health?
- **What advances does it highlight?**
  Chronic respiratory acidosis can cause bone demineralization, renal calcification, perinatal developmental abnormalities, systemic inflammation and impairments in cognitive function and visuomotor skills and can produce cerebral acidosis, potentially inducing sustained alterations in cerebral function.

## 1 INTRODUCTION

**FIGURE 1:**
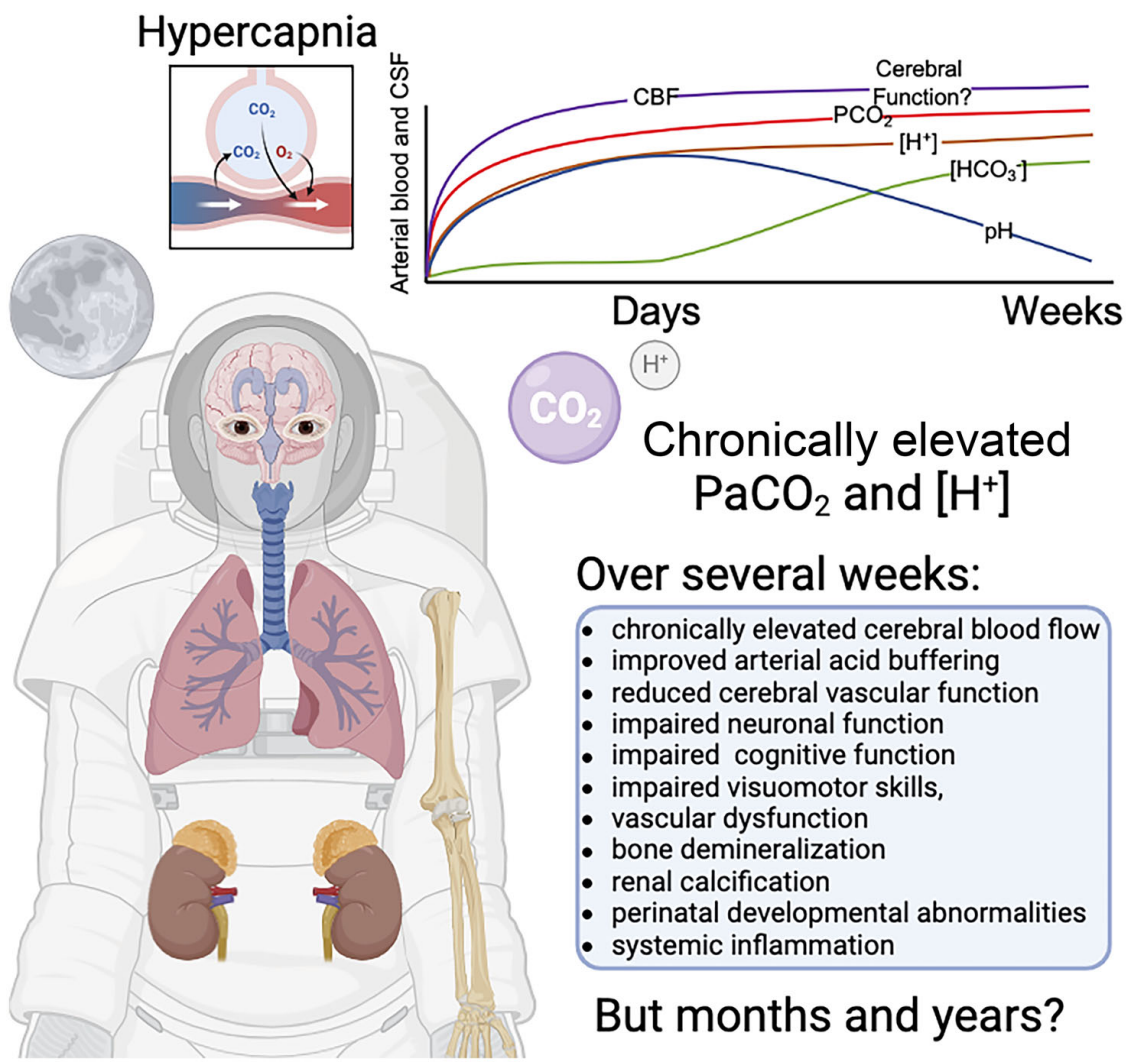
The effects of long‐term elevations in $P_{\mathrm{aC}O_{2}}$.

A powerful physiological stimulus, CO_2_ elicits profound effects on a number of integrated physiological systems. The physiological effects of CO_2_ in the body have been known for over a century (Hill, 1896; Pike, 1918; Roy & Sherrington, 1890). A common focus of much physiological research has been the consequences of elevated partial pressures of arterial CO_2_ ($P_{\mathrm{aC}O_{2}}$; i.e., hypercapnia) over an acute time frame, both for its utility as an investigational tool (e.g., tests of ventilatory chemoreflex sensitivity and cerebrovascular reactivity) and for its utility as a clinically relevant sequela (i.e., respiratory acidosis is a symptom of many pathophysiological states). The physiological effects of longer exposures (hours to days) are also pertinent to environmental scenarios, such as industrial and military contexts, wildfire exposure, mining and agriculture. The physiological consequences of chronic exposure to elevated CO_2_ include systemic inflammation, changes in bone mineral density, renal calcification, behavioural changes, vascular dysfunction and cognitive impairment (Jacobson et al., 2019) (Figure 1). Indeed, lifetime CO_2_ exposure is increasingly more relevant, given a number of considerations, including: (1) rising global atmospheric concentrations [typically ∼400 parts per million (ppm) depending on location and season, and projected to rise to 1000 ppm by the end of the century (Prather et al., 2014)]; (2) the requirements for safe interplanetary travel, given the potential impacts on cognition and troubleshooting during anomalous technical malfunction (Beard, 2020); and (3) pathophysiological respiratory acidosis and concomitant renal compensation.

Extended exposure to moderately elevated CO_2_ is common aboard the International Space Station. Indeed, CO_2_ can reach concentrations 10 times greater than in the terrestrial atmosphere (Law et al., 2014; NASA, 2023a), owing to metabolic production from the crew and the limited capacity for CO_2_ scrubbing. There are several systems regulating International Space Station CO_2_ concentrations (NASA, 2015), whereby NASA aims to control steady‐state cabin CO_2_ to a maximum of ∼3–4 mmHg inspired partial pressure of CO_2_ ($P_{I,CO_{2}}$; ∼0.4% of 760 mmHg barometric pressure; for fraction and pressure conversions, see Table 1) (NASA, 2023a). However, in the event of a transient and extreme rise in ambient CO_2_, such as those which occurred aboard *Apollo 13* in 1970 or *Soyuz 23* in 1976 (NASA, 2023a), the physiological state of astronauts is of critical importance to their performance and survival. Even without such an event, work in confined areas, where air stagnation can occur, could lead to local CO_2_ accumulation. Given these points, interplanetary travel to and subsequent colonization of the Moon, Mars and beyond will require careful consideration of the physiological effects of chronic ‘low‐level’ CO_2_ exposure. NASA's *Artemis* Lunar exploration programme will establish a base in lunar orbit, from which further flights to the lunar south pole and even Mars are planned. *Artemis 2*, the first crewed mission, will last 10 days. *Artemis 3*, the crewed moon surface landing, will include a flight lasting 30 days, 1 week on the surface and a return flight of 30 days. The *Artemis 4* mission involves the establishment of a crew on the Lunar *Gateway*, the first lunar space station. These endeavours necessitate safe occupation of confined spaces, including tight atmospheric regulation of inspired gases.

**TABLE 1:** Conversion chart for pressure and fraction of inspired CO2, calculated for barometric pressures of 760 and 400 mmHg. Notable CO2 concentrations/pressures (at sea‐level barometric pressure) are highlighted.

	$P_{I,CO_{2}}$ at 760 mmHg barometric pressure		Concentration			$P_{I,CO_{2}}$ at 400 mmHg barometric pressure	
Notable CO_2_	(mmHg)	(kPa)	(%)	(ppm)	($F_{I,CO_{2}}$)	(mmHg)	(kPa)
Atmospheric average in 1960	0.23	0.031	0.032	320	0.00032	0.11	0.015
Current atmospheric average (2024)	0.29	0.04	0.04	400	0.0004	0.14	0.02
NASA nominal vehicle/habitat $P_{I,CO_{2}}$ (NASA, 2023b),^a^ ACGIH TLV (ACGIH, 2023), NIOSH‐REL (NIOSH, 2023)	3.57	0.48	0.5	5000	0.005	1.77	0.24
NASA spacesuit $P_{I,CO_{2}}$ indefinite exposure limit (NASA, 2023b)^b^	4.28	0.57	0.6	6000	0.006	2.12	0.28
RCP8.5 (Prather et al., 2014)^c^	7.13	0.95	1	10,000	0.01	3.53	0.47
NASA spacesuit $P_{I,CO_{2}}$ ‘do not exceed limit’ (NASA, 2023b)^d^	14.26	1.9	2	20,000	0.02	10.41	1.39
ACGIH‐STEL (ACGIH, 2023)	21.39	2.85	3	30,000	0.03	10.59	1.41
NIOSH‐IDLH (NIOSH, 2023)	28.52	3.8	4	40,000	0.04	14.12	1.88
$F_{I,CO_{2}}$ commonly used in research in acute exposures	35.65	4.75	5	50,000	0.05	17.65	2.35
	39.22	5.23	5.5	55,000	0.055	19.42	2.59
	42.78	5.7	6	60,000	0.06	21.18	2.82
	46.35	6.18	6.5	65,000	0.065	22.95	3.06
	49.91	6.65	7	70,000	0.07	24.71	3.29
$F_{I,CO_{2}}$ above which extended exposures will be likely to result in death^e^	78.43	10.46	11	110,000	0.11	38.83	5.18
	142.6	19.01	20	200,000	0.2	70.6	9.41
	178.25	23.76	25	250,000	0.25	88.25	11.77
$F_{I,CO_{2}}$ used acutely by Meduna (1950) for psychotherapy	213.9	28.52	30	300,000	0.3	105.9	14.12

Abbreviations: ACGIH, American Conference of Governmental Industrial Hygienists; $F_{I,CO_{2}}$, fractional inspired CO_2_; IDLH, immediately dangerous to life or health; NIOSH, National Institute for Occupational Safety and Health; $P_{I,CO_{2}}$, inspired partial pressure of CO_2_; REL, recommended exposure limits; STEL, short‐term exposure limit; TLV, threshold limit values.

^a^ ‘The system shall limit the average 1‐h CO_2_ partial pressure (ppCO2) in the habitable volume to no more than 3 mmHg’ (NASA, 2023b).

^b^ $P_{I,CO_{2}}$ ≤ 4.0 mmHg allowable indefinitely [see NASA (2019): table 11.3‐1].

^c^ Projected atmospheric CO_2_ ppm due to expected carbon emissions over the next ∼80 years.

^d^ $P_{I,CO_{2}}$ > 15.0 mmHg do not exceed [see NASA (2019): table 11.3‐1].

^e^ Short durations of very high $F_{I,CO_{2}}$ are survivable with discomfort; however, exposures to such concentrations without respite will result in respiratory and circulatory arrest eventually.

Herein, we summarize the cerebral impacts of hypercapnia over chronic exposures (days to months) and, for contextualization, we also include discussion of the respiratory and metabolic compensations. We draw on literature from preclinical, physiological and clinical work in humans. Prolonged and chronic hypercapnia is less commonly used in physiological research than acute hypercapnia, hence our understanding of the cerebral consequences of longer exposures relies mostly on animal and clinical research.

### 1.1 Blood gases and acid–base balance

Fundamentally, the danger of excess molecular concentrations of CO_2_ in tissues stems, for the most part, from the relationship between CO_2_ and hydrogen ion concentration ([H^+^]). Acidosis (i.e., elevated [H^+^]) alters the molecular form of many proteins, reducing enzymatic activity, causing apoptosis (Lagadic‐Gossmann et al., 2004) and necrosis (Kraig et al., 1987) if severe enough. Proteins are susceptible to physicochemical deformation and thereby impaired protein function (Broda et al., 1959; Kraig et al., 1987), due to an altered milieu of free protons, negatively‐affecting the metabolic rate.

The traditional approach to acid–base balance expresses pH and [H^+^] as being determined predominantly by [HCO_3_^−^] and $P_{CO_{2}}$ (Henderson, 1908; Schwartz & Relman, 1963). An alternative approach was proposed by Stewart (1983), who suggested that [H^+^] is determined by the physicochemical properties of ions, in accordance with the laws of mass action, conservation of mass and conservation of charge (Stewart, 1983). Specifically, Stewart's approach realizes [H^+^] with the application of six equilibrium equations concerning three independent variables, which determine six dependent variables including [H^+^]. For comparison of the traditional and Stewart approaches, the reader is directed to earlier reviews (Corey, 2003; Kimura et al., 2018; Kurtz et al., 2008; Morgan, 2009; Rastegar, 2009). The present review treats pH in terms of the traditional model rather than Stewart's model.

The history of blood gas analysis is rife with concepts and terms aimed at complete considerations of acid–base status, for the most part conceived in order to aid diagnoses of acid–base disorders. Exposition of these terms is far beyond the remit of this review; however, interested readers should consider the historical series by Severinghaus, Astrup and Honda (Astrup & Severinghaus, 1986; Severinghaus & Astrup, 1985a, 1985b, 1986a, 1986b, 1986c, 1986d; Severinghaus & Honda, 1987). Those with particular interest in the cerebral vasculature and blood gases should also see Caldwell et al. (2021) and Hoiland et al. (2019) for reviews of cerebrovascular function in metabolic and respiratory acid–base perturbations, respectively.

Sustained hypercapnia increases peripheral tissue oxygenation in humans by right shifting the oxyhaemoglobin desaturation curve (Bohr effect), which therefore increases offload of O_2_ at the tissues (Akça et al., 2002; Fleischmann et al., 2006), but also mildly decreases resting arterial O_2_ saturation. Hypercapnia may also increase O_2_ carriage in the blood via increased haemoglobin concentration and, possibly, by improved ventilation–perfusion matching in the lungs, as demonstrated in dogs [at least over 15 min (Torbati et al., 1998)]. Although there is complementary evidence in humans that CO_2_ might mediate some elevations in haemoglobin concentration via splenic contraction (Richardson et al., 2012), it has yet to be established that hypercapnia per se is responsible, and that this effect is not merely a consequence of the increase in sympathetic nervous activity that hypercapnia promotes (Sotiridis et al., 2024).

### 1.2 Metabolic compensations in the arterial blood and CSF

Owing to the continuous metabolic generation of acids and the free glomerular filtration of bicarbonate, the renal tubules normally mediate the reabsorption of filtered bicarbonate and concomitant secretion and excretion of H^+^ in urine, maintaining systemic pH. These regulatory mechanisms are mediated by both luminal facing (i.e., extracellular) and intracellular carbonic anhydrase (CA) in renal tubule intercalated cells. Normally, both isoforms of CA co‐ordinate the reabsorption of filtered bicarbonate from the tubule lumen for ultimate reabsorption into the systemic circulation. Importantly, the altered behaviour of CA in renal tubules in sustained acid–base perturbations allows for appropriate changes in the renal handling of bicarbonate and H^+^, facilitating compensatory responses. These integrative respiratory–renal mechanisms mediate blood gas and acid–base homeostasis in sustained, environmentally induced blood gas challenges.

**FIGURE 2:**
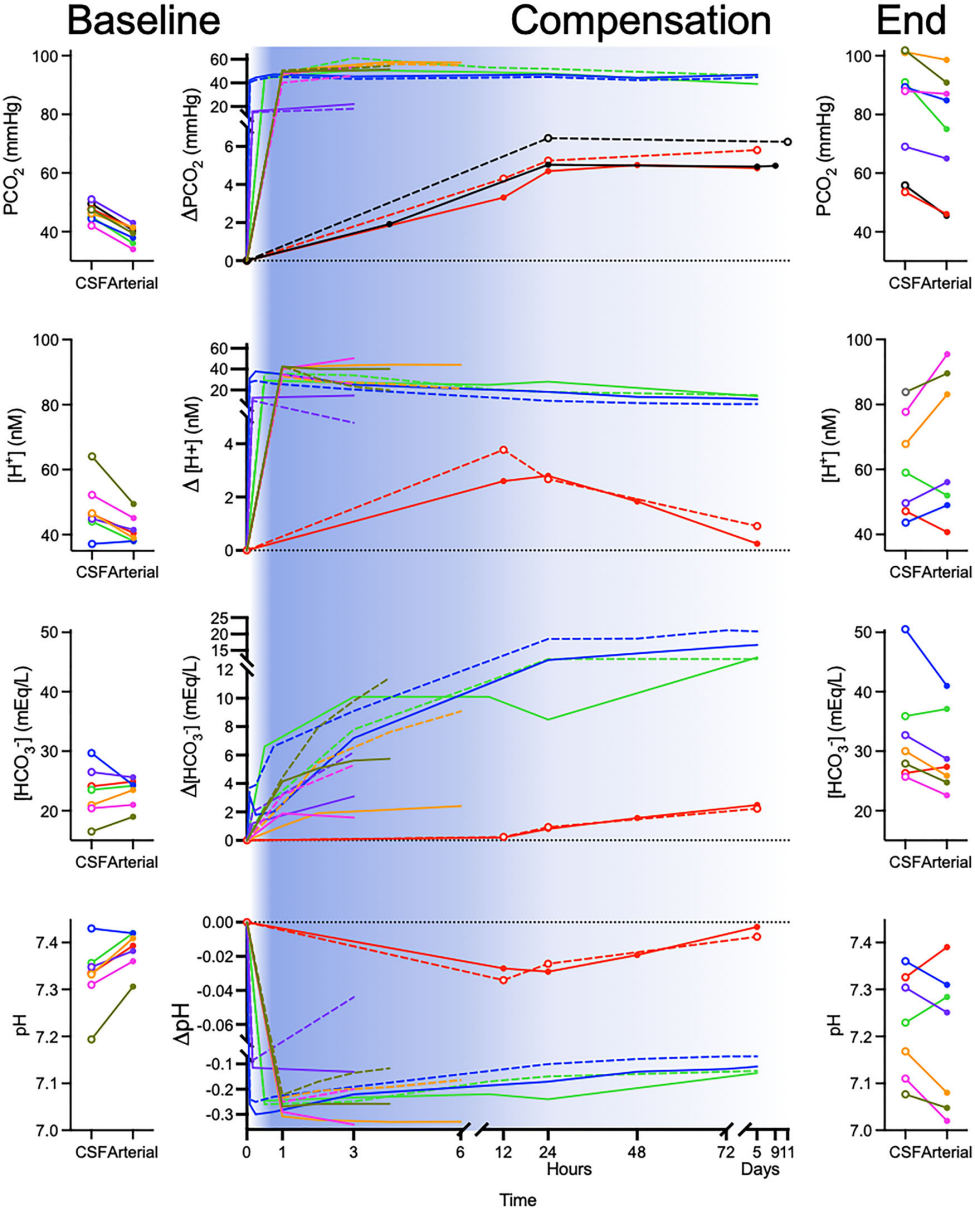
Arterial and CSF acid–base compensation during experimental chronic respiratory acidosis. Each colour represents a single study. Acid–base changes from baseline in arterial blood (continuous lines) and CSF (dashed lines), in humans (red and black), rats (blue) and dogs (green, purple, pink, yellow and dark green) exposed to hypercapnia for ≤4 days. Sampling time points have been highlighted in humans via symbols, closed for arterial and open for CSF. Arterial blood acid–base compensation is approximated via a colour gradient, where the change from blue to clear indicates the likely range of times that renal retention of [HCO_3_^−^] might begin to have a notable effect on countering the elevation of [H^+^]. Note the extreme difference between animal and human models in the degree of $P_{\mathrm{aC}O_{2}}$ increase. Clark et al. (1971) exposed two groups of four adult males to 30 mmHg ambient $P_{CO_{2}}$ for 5 (*n* = 4) and 11 days (*n* = 4). Bleich et al. (1964) exposed 35 dogs to 12% CO_2_ (∼80 mmHg $P_{I,CO_{2}}$), and 6–10 dogs were assessed per time point. Messeter and Siesjö (1971) exposed rats to 11% CO_2_ (four or five rats per time point). Arieff et al. (1976), Wichser and Kazemi (1975) and Kazemi et al. (1967) used 10% CO_2_ in groups of five, six and eight dogs, respectively. Nattie and Edwards (1981) exposed groups of six puppies to 8% CO_2_. It is unclear why Messeter and Siesjö’s (1971) rats began from a mild cerebral alkalosis, although it might be that rats have slightly higher CSF [HCO_3_^−^] at rest than humans (Kaällquist, 1970; Reed et al., 1967). Likewise, it is unclear why Wichser and Kazemi's (1975) dogs began from a more pronounced cerebral acidosis with no arterial acidosis. The finding that most animal studies seem to have demonstrated an early rise in CSF [HCO_3_^−^] that is greater than the rise in arterial [HCO_3_^−^], whereas the only two humans studies did not, might reflect species differences in blood–brain barrier active [HCO_3_^−^] transport (see further discussion by Kazemi & Johnson, 1986), or differences in the magnitude of hypercapnia (+4 mmHg $P_{\mathrm{aC}O_{2}}$ in humans and +40–60 mmHg $P_{\mathrm{aC}O_{2}}$ in animals), which affects ionic transport, or it could simply be that the first sample time point in the study by Clark et al. (1971) missed a transient rise in [H^+^] and [HCO_3_^−^] prior to 12 h. For clarity of presentation, data from Clark et al.’s (1971) 5‐day exposure has only been used where CSF and arterial sampling were time matched. Additionally, all of the presented animal studies used $P_{I,CO_{2}}$ high enough to reverse the normal CSF–arterial $P_{CO_{2}}$ gradient, thereby forcing an uncompensable cerebral acidosis, whereas the human studies used stimuli closer to that which might be experienced aboard spaceflight, for example, and found that both the arterial and CSF compartments were able to compensate to a degree. Several studies have assessed the alveolar $P_{CO_{2}}$, $P_{\mathrm{aC}O_{2}}$ and other variables during exposure to CO_2_, but we have focused here on only those studies in otherwise healthy animals/humans (e.g., Roncoroni et al., 1970 assessed 4 h of +40–50 mmHg $P_{\mathrm{aC}O_{2}}$ in two decerebrate patients with no spontaneous respiratory drive), which also measured CSF acid–base and where hypercapnia was constant [e.g., in the study by Jennings & Davidson, 1984, chronic hypercapnia (5% CO_2_) was imposed in dogs for 2, 5, 4 and 10 days, but each bout was separated by 1–2 days of normocapnia]. Abbreviations: CSF, cerebrospinal fluid. [HCO_3_^−^], bicarbonate ion concentration. [H^+^], hydrogen ion concentration. $P_{CO_{2}}$, partial pressure of carbon dioxide.

As illustrated in Figure 2, the acid–base response to chronic hypercapnia can be characterized into two periods; (1) initial exposure, wherein $P_{\mathrm{aC}O_{2}}$ is elevated, [HCO_3_^−^] has yet to rise via renal retention and buffer the insult, hence pH is lower than in normocapnia (i.e., respiratory acidosis); and (2) compensation, where $P_{\mathrm{aC}O_{2}}$ either remains above normal or is somewhat normalized (resulting from a respiratory chemoreflex), and [HCO_3_^−^] has risen via renal retention such that pH is normalized or near normal (Guillerm & Radziszewski, 1979; Schaefer et al., 1963; Wade et al., 2000), depending on the magnitude. The duration and persistence of the uncompensated phase of exposure and the magnitude changes in $P_{\mathrm{aC}O_{2}}$, [H^+^] and [HCO_3_^−^] are all obviously dependent on the onset time and magnitude of the $P_{I,CO_{2}}$ stimulus; however, at least with a fractional inspired CO_2_ ($F_{I,CO_{2}}$) of ∼0.015–0.02 (10.7–14.3 mmHg $P_{I,CO_{2}}$), compensation seems to occur within ∼15–23 days in humans (Guillerm & Radziszewski, 1979; Schaefer, 1963; Schaefer et al., 1963), and in 0.03–0.04 $F_{I,CO_{2}}$, compensation can occur in ∼2–5 days (Clark et al., 1971; Glatte et al., 1967; Nicolaysen et al., 1989). [Achieving compensation might be more rapid in animals, such as dogs and rodents (Polak et al., 1961; Raichle & Stone, 1971; Schwartz et al., 1961; Wade et al., 2000), although greater hypercapnia is sometimes used in these studies.] However, failure to compensate arterial pH during 44 days of 0.01 $F_{I,CO_{2}}$ (∼7 mmHg $P_{I,CO_{2}}$) has also been reported (Pingree, 1977). Figure 2 depicts arterial acid–base compensation from studies in which arterial and CSF values have been measured simultaneously during chronic hypercapnia in healthy humans and animals.

The compensation phase involves not only renal retention of bicarbonate and excretion of H^+^ and NH_4_^+^, but also retention of magnesium, inorganic phosphates and calcium (Gray et al., 1973). The latter relates to a decrease in bone calcium content owing to the CO_2_ sequestering into carbon bone buffers and subsequent bone demineralization (Drummer et al., 1998; Schaefer et al., 1980, 1979). Bone CO_2_ buffering and the associated bone calcium loss then also impacts renal function through calcification. The impact of chronically elevated CO_2_ on both bone and renal function is likely not to be as significant as the effect of weightlessness (Smith et al., 2014); nonetheless, it could be significant enough to warrant consideration for long‐term spaceflight.

Interstitial fluid and CSF surround the brain, providing it with nourishment and buoyancy and functioning as the main conduit of metabolic waste removal and for transfer of growth and neuronal maintenance factors (Spector et al., 2015). These fluids are relatively protein poor, but they contain HCO_3_^−^ and can therefore buffer changes in $P_{CO_{2}}$ within a fairly narrow range (Fencl, 1986; Siesjö, 1972), particularly during acute (Fisher & Christianson, 1963) and chronic respiratory alkalosis (Severinghaus et al., 1963). Although CSF [HCO_3_^−^] is slower to change than CSF $P_{CO_{2}}$ (Bleich et al., 1964; Messeter & Siesjö, 1971), it does, nevertheless, change over the course of hours and can eventually reach concentrations sufficient almost to normalize CSF pH when CSF $P_{CO_{2}}$ is elevated. The CSF is more acidotic than arterial blood in normal circumstances (Fencl, 1986; Mitchell et al., 1965a) simply owing to metabolism, and this is also the case in chronic respiratory acidosis (Clark et al., 1971). However, decreases in arterial pH seem less well buffered in the CSF (Clark et al., 1971), at least in pathological chronic respiratory acidosis (Fencl, 1986; Merwarth et al., 1961; Mitchell et al., 1965b; Roncoroni et al., 1970; Siesjö, 1972; Skinhøj, 1968). There is reason to expect differences between cisternal and lumbar cerebrospinal fluid $P_{CO_{2}}$, pH and [HCO_3_^−^] in both health and chronic hypercapnic disease (Fisher & Christianson, 1963), which is relevant to assessment of CSF acid–base during relatively short‐duration changes in arterial acid–base status, but probably irrelevant during chronic respiratory acidosis. In contrast, in experimental animal models of multiple days of hypercapnia (Bleich et al., 1964; Messeter & Siesjö, 1971) and in two decerebrate normocapnic humans (with no spontaneous ventilatory drive) during 4 h of hypercapnia (Roncoroni et al., 1970), changes in CSF pH seem similar to the arterial pH change initially; however, thereafter the CSF pH is more swiftly normalized than arterial pH owing to rapid increases in CSF [HCO_3_^−^]. The differences in CSF pH regulation between animal and human studies (decerebrate case studies aside) are probably explained by the magnitude of $P_{\mathrm{aC}O_{2}}$ elevation used. For example, animal studies often impose non‐physiological $P_{\mathrm{aC}O_{2}}$ elevations of +40 mmHg, reversing the normal CSF–arterial $P_{CO_{2}}$ gradient by far and also surpassing the capacity for increases in cerebral blood flow (CBF) to aid the normalization of the CSF–arterial $P_{CO_{2}}$ gradient via increases in CO_2_ washout. As such, in humans with a chronic respiratory acidosis of physiological magnitude, the decrease in CSF pH is likely either to match or to exceed the arterial decrease (Mitchell et al., 1965b).

**FIGURE 3:**
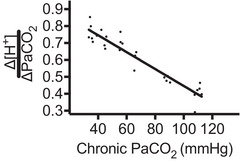
Acid buffering with chronic respiratory acidosis. As steady‐state $P_{\mathrm{aC}O_{2}}$ increases, the hydrogen ion response to acute changes in $P_{\mathrm{aC}O_{2}}$ decreases. This is the result of the chronic compensatory increase in [HCO_3_^−^]. These data were redrawn from Goldstein et al. (1971). Although base compensation may take longer to occur in the CSF than in the arterial blood during chronic respiratory acidosis, if and when it does eventually occur, these changes in hydrogen ion ‘reactivity’ to CSF $P_{CO_{2}}$ will likewise present in the CSF. Abbreviations: [H^+^], hydrogen ion concentration. $P_{\mathrm{aC}O_{2}}$, arterial partial pressure of carbon dioxide.

Following chronic hypercapnia, there is an increase in acute arterial buffering capacity owing to the elevation in [HCO_3_^−^] (following renal compensation) (Goldstein et al., 1971; Schwartz et al., 1965). This means that the slope of the change in [H^+^] with an acute change in $P_{\mathrm{aC}O_{2}}$ is less steep after compensation than before it. This has also been demonstrated in humans with chronic lung disease (van Ypersele de Strihou et al., 1966). This means that after compensation in the arterial circulation, acute‐on‐chronic CO_2_ insults produce a relatively lesser degree of acidosis. Figure 3 depicts data from animal work showing the improvement in acid buffering with compensated chronic acidosis.

**FIGURE 4:**
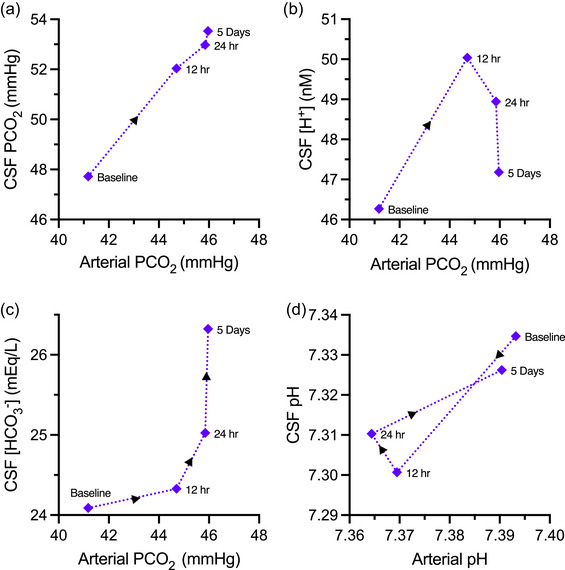
Cerebrospinal fluid acid–base compensation during 5 days of hypercapnia in healthy humans. The figure depicts the changes over time in CSF $P_{CO_{2}}$ (a) with mild respiratory acidosis, which acutely elevates [H^+^] (b) and slowly elevates [HCO_3_^−^] (c). Note that although CSF pH is somewhat normalized (d), it remains slightly acidotic. At this new acid–base steady state, with an elevated [HCO_3_^−^], an acute‐on‐chronic CO_2_ insult would be buffered more effectively than prior to chronic acidosis, resulting in a lesser pH change. We speculate that this might result in a reduced effect of acute $P_{CO_{2}}$ changes on the cerebrovasculature. Black arrows indicate the direction of changes over time. Data are from Clark et al. (1971), 5 days of 30 mmHg ambient $P_{CO_{2}}$. Abbreviations: CSF, cerebrospinal fluid. [HCO_3_^−^], bicarbonate ion concentration. [H^+^], hydrogen ion concentration. $P_{CO_{2}}$, partial pressure of carbon dioxide.

Acute‐on‐chronic $P_{CO_{2}}$ challenges following CSF pH compensation would produce a lesser CSF [H^+^] change than in normocapnia (Raichle & Stone, 1971). As mentioned above, return to normocapnia following prolonged hypocapnia results in hyperaemic overshoot, which is speculated to be attributable to reduced CSF buffering capacity after extended [HCO_3_^−^] loss. Conversely, if extended hypercapnia produces a greater intracerebral buffering capacity (i.e., greater [HCO_3_^−^]), this could present as a reduced cerebrovascular reactivity (CVR) to CO_2_. Although such a reduction in CVR might, at first glance, be interpreted as an impairment, it could, in fact, represent appropriate adaptation to the environmental stressor. This is relevant in the context of prolonged and chronic respiratory acidosis because of the improved acid buffering that ensues. In the context of respiratory acidosis in health (i.e., prolonged living in an enclosed environment, e.g., spaceflight) this might mean that CVR is reduced from the terrestrial normocapnic baseline, but despite this reduction, cerebral acid–base status is not negatively affected. In the context of respiratory acidosis in pathology (i.e., chronic pulmonary disease), increased buffering‐related reductions in CVR might represent appropriate CVR, which might otherwise be attributed to pathological impairment in the cerebrovasculature (obviously, vascular dysfunction is a major contributor to and consequence of many chronic diseases and will also explain many changes in CVR). See Figure 3 for arterial acid buffering during chronic respiratory acidosis and Figure 4 for CSF acid–base compensation and subsequent pH resetting with chronic respiratory acidosis in healthy humans.

## 2 PROLONGED HYPERCAPNIA, PRIOR TO METABOLIC COMPENSATION

Although not as common in research as acute hypercapnia, prolonged moderate hypercapnia (i.e., >1 h, <24 h) represents a unique stimulus, given the possibility of problems associated with elevated CO_2_ in the modern workplace environment. Regulation of indoor CO_2_ concentration is a crucial element of safe working environments (Jacobson et al., 2019), because even 1000 ppm [well below the American Conference of Governmental Industrial Hygienists (ACGIH) threshold limit values (TLV) (ACGIH, 2023) and National Institute for Occupational Health and Safety (NIOSH) recommended exposure limit (REL) (NIOSH, 2023) of 5000 ppm] has been found to impair cognition and decision‐making after only 2 h (Cedeño Laurent et al., 2021; Du et al., 2020; Satish et al., 2012; Zhang et al., 2021) and can elicit headaches (James, 2007). The mechanisms of such effects of hypercapnia on cognition might be related to increases in intranuclear and free cytosolic Ca^2+^ and cortical membrane lipid peroxidation (Fritz et al., 2024). Acid‐sensing ion channels are proton‐sensitive receptors and are extensively expressed throughout the brain in regions related to anxiety behaviour, locomotion, motor learning, fear and negative emotions, spatial memory, addictive behaviour and pain perception (Storozhuk et al., 2021), and have numerous roles in synaptic plasticity, ischaemic neuronal injury and mechano‐sensation, being crucial for CO_2_‐related vasodilatation (reviewed by Kweon & Suh, 2013; Zha et al., 2022). The nuances of acid‐sensing ion channels are beyond the scope of this review. Whatever the fundamental mechanisms underlying hypercapnia‐related cognitive impairments, this is undoubtedly a concern for long‐duration spaceflight.

Research in humans investigating prolonged hypercapnia often focuses on the effects in the setting of combined sedentary behaviour (which is likely to confounds interpretation of the effects of CO_2_ per se; e.g., Headid et al., 2020; Park et al., 2022). Such studies also often use very mild CO_2_ stimuli (∼1% CO_2_), which appropriately simulates many workspace environments. However, a major issue with such studies is that measurement error and physiological variability necessitate using a powerful experimental design to elicit detectable increases in $P_{\mathrm{aC}O_{2}}$, at least according to Forster et al. (1982), who detected an increase in $P_{\mathrm{aC}O_{2}}$ above baseline only when $P_{I,CO_{2}}$ was ≥21 torr (∼2.9%). There is disagreement regarding whether small (<1%) hypercapnic stimuli change $P_{\mathrm{aC}O_{2}}$ or whether ventilatory responses are able to combat the challenge to maintain a state of isocapnic hyperpnoea [e.g., Forster et al. (1982) vs. Ellingsen et al. (1987)]. This is mostly because interpretation of ventilatory sensitivity becomes more challenging with smaller and smaller $\Delta P_{I,CO_{2}}$, because ΔV˙E/ΔV˙EΔPaCO2ΔPaCO2 approaches infinity and becomes indeterminable (Dejours et al., 1965; Dempsey, 1976). Nevertheless, there is utility in both low (*viz*. ecological validity) and high (*viz*. greater signal‐to‐noise ratio) CO_2_ stimulus approaches.

Additionally, a slower rate of rise might influence the final magnitude of cardiorespiratory changes, that is, a rise to 6% across 5 h might produce a lesser increase in ventilation than across 1–2 h (Sidorov & Sulimo‐Samuyllo, 1977). This might be attributable to a greater time for metabolic buffering, allowing a more steadily maintained pH.

### 2.1 Cerebral blood flow prior to metabolic compensation

#### 2.1.1 Animals

Although the effects of acute (minutes) hypercapnia on CBF are well established, there remains some degree of dispute regarding whether CBF remains elevated, declines or increases with longer exposures. Hino et al. (2000) assessed CBF and the cerebral metabolic rate of O_2_ (CMRO_2_) in newborn lambs during and after 6 h of hypercapnia (∼78 torr $P_{\mathrm{aC}O_{2}}$). Although CBF was initially elevated far above baseline, after 6 h the CBF was only mildly elevated and returned to baseline following return to normocapnia (Hino et al., 2000). During hypercapnia, cerebral delivery of O_2_ (CDO_2_) increases owing to the increase in CBF, while the oxygen extraction fraction (OEF) is decreased, such that CMRO_2_ continues unhindered (Hino et al., 2000). Contrastingly, during hypocapnia, the decrease in CBF forces a concomitant decrease in CDO_2_, which is compensated for by an increase in OEF, again such that CMRO_2_ is unchanged (Gleason et al., 1989). Finally, the acute increase in CBF with hypercapnia remained by the 6 h time point, whereas the decrease with hypocapnia was fully corrected after 6 h, indicating probable CSF pH correction or CBF responding to decreased CDO_2_ via cerebral hypoxic vasodilatation (Carr et al., 2024). These changes are likely to be explained by active transport of [HCO_3_^−^] into the CSF, which seems to occur more rapidly in animal models of respiratory acidosis (Kazemi & Johnson, 1986), possibly owing to differences in the magnitude of the CO_2_ stimuli. Najarian et al. (2000) found in hypercapnic pigs that CBF increases initially, declined around 3 h, then gradually increased again after 6 and 8 h. Najarian et al. (2000) found that this second hyperaemia was an endothelial nitric oxide synthase‐related response, dependent on interaction between ATP‐dependent potassium channels and prostaglandins. Whether this CBF profile occurs in humans is entirely unexplored.

As discussed above in Section 1.2, during prolonged hypocapnia, loss of brain extracellular [HCO_3_^−^] reduces the CSF buffering capacity for subsequent changes in pH, such that the return of normocapnia then causes CSF pH overshoot, and CBF increases above the normocapnic baseline (Curley et al., 2010). This is relevant in the context of prolonged and chronic respiratory acidosis because of the improved acid buffering that ensues. Following CSF compensation to chronic respiratory acidosis, in the case of acute rise in $P_{\mathrm{aC}O_{2}}$, the elevated CSF [HCO_3_^−^] allows for a less severe increase from baseline CSF [H^+^] and therefore a less severe increase in CBF and intracerebral pressure (ICP). However, in the case of an acute drop in $P_{\mathrm{aC}O_{2}}$, the alkaline shift in the CSF is more pronounced, because the elevated [HCO_3_^−^] is ‘trapped’, as it were (Skinhøj, 1968). In spaceflight, where chronic mild respiratory acidosis might occur, altered arterial and CSF acid buffering will have implications for small changes in $P_{\mathrm{aC}O_{2}}$ during exercise and sleep etc.

## 3 CHRONIC HYPERCAPNIA, FOLLOWING METABOLIC COMPENSATION

Studies in adult humans that would best simulate long‐term spaceflight conditions of chronic hypercapnia [i.e., including longer exposures, with varied activity levels (e.g., Consolazio et al., 1947; Cutler et al., 1964; Faucett & Newman., 1953; Schaefer, 1963, 1979; Schaefer & Baker III, 1969; Sinclair et al., 1971)] have fallen into relative obscurity. Nonetheless, these pioneering studies demonstrated that healthy humans can productively live, work and even exercise moderately in environments up to 0.054 $F_{I,CO_{2}}$ (∼41 mmHg $P_{I,CO_{2}}$ at 1 atm) for any time from 3 to 43 days, although, of course, not without some degree of discomfort. Visuomotor performance can be impaired by CO_2_ concentrations as low as 1.2% (Manzey & Lorenz, 1998).

As mentioned in Section 2, insufficient CO_2_ stimuli (below some threshold) may fail to produce measurable $P_{\mathrm{aC}O_{2}}$ changes. The same issue befalls many long‐duration studies. For example, exposure to a ∼0.5% CO_2_ environment (∼3.6 mmHg) did not increase arterialized‐capillary $P_{CO_{2}}$ (Laurie et al., 2020) after 1 or even 30 days of exposure, although it did increase [HCO_3_^−^]. This lack of $P_{CO_{2}}$ derangement does not necessarily indicate a failed experiment, because the stimulus was sufficient to elicit blood buffering and renal responses, which would simulate those effects of mild CO_2_ exposure during spaceflight (Leacy et al., 2020).

### 3.1 Cerebral blood flow following metabolic compensation

#### 3.1.1 Animals

**FIGURE 5:**
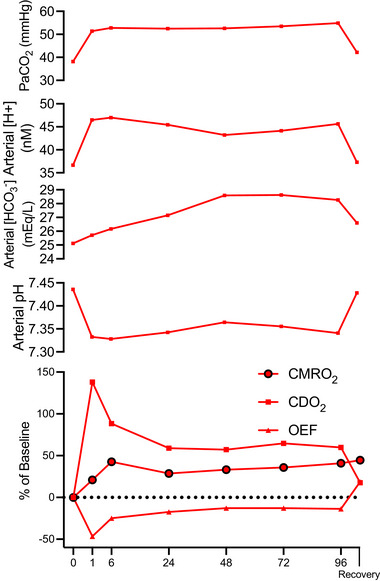
Arterial acid–base and changes in cerebral metabolism during 96 h of hypercapnia in awake adult sheep. During hypercapnia, CDO_2_ increases owing to the increase in cerebral blood flow, while the OEF is decreased, such that CMRO_2_ continues unhindered. Indeed, by the 6 h time point, CMRO_2_ was significantly greater than baseline. Abbreviations: BL, baseline; CDO_2_, cerebral delivery of O_2_ (squares); CMRO_2_, cerebral metabolic rate of O_2_ (circles); [H^+^], hydrogen ion concentration; OEF, oxygen extraction fraction (triangles). Time points were at baseline; $P_{CO_{2}}$, partial pressure of carbon dioxide; 1, 6, 24, 48, 72 and 96 h during hypercapnia; and 3 h following recovery (Recovery) to room air in conscious adult sheep. Data were redrawn from Yang and Krasney (1995). [HCO_3_^−^], bicarbonate ion concentration.

Yang and Krasney (1995) submitted conscious adult sheep to 96 h of +15 mmHg $P_{\mathrm{aC}O_{2}}$ (see Figure 5). During this long exposure, in similar manner to acute hypercapnia, CBF was elevated and OEF decreased, serving the maintenance of CMRO_2_. From 6 h onwards, cerebral metabolism was increased from 3.5 ± 1.0 mL/min/100 g at baseline to ∼5.0 ± 1.3 mL/min/100 g, even extending into room air recovery and outlasting the return to near‐normal values of both CDO_2_ and OEF. These animals presented with modestly increased brain tissue water content in the frontal and occipital lobes, indicating a degree of cerebral oedema and/or increased blood–brain barrier permeability. In rhesus monkeys, following 5 days in ∼45 mmHg $P_{I,CO_{2}}$ (∼6% CO_2_), the relationships between carotid blood velocity and mean arterial pressure and between blood velocity and arterial $P_{CO_{2}}$ were blunted [mean arterial pressure was altered via exsanguination and infusion of the adrenergic α_1_ agonist aramine, and CVR was tested via various values of $F_{I,CO_{2}}$ (0.06, 0.09 and 0.12) (Raichle & Stone, 1971)].

#### 3.1.2 Humans

**FIGURE 6:**
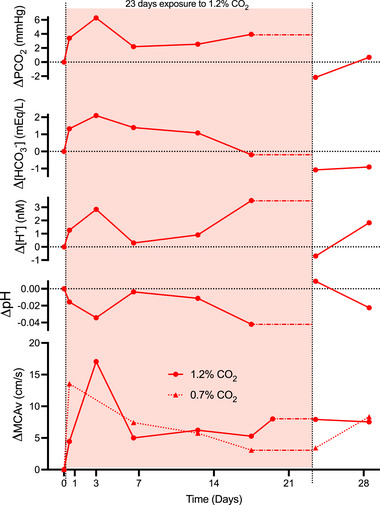
Acid–base and middle cerebral artery blood velocity during 23 days of experimental respiratory acidosis in healthy humans. Sliwka et al. (1998) exposed four healthy male adults to 1.2% CO_2_ for 23 days, during which arterialized blood samples were taken and transcranial Doppler ultrasound was used to assess middle cerebral artery blood velocity. The same four individuals were also exposed to 23 days of 0.7% CO_2_. The middle cerebral artery blood velocity (MCAv) was assessed (dotted line), but no blood samples were taken. The first time point post‐hypercapnia was during normal room air breathing and incurred a slight hypocapnia, presumably owing to some ventilatory facilitation following the removal of the CO_2_ stimulus; therefore, the dashed lines between the last time point during hypercapnia and the end of imposed hypercapnia have been inferred from the last hypercapnia time point. It is unclear why arterial [HCO_3_^−^] did not remain elevated in these individuals towards the end of 23 days, despite the continued respiratory acidosis. Abbreviations: [HCO_3_^−^], bicarbonate ion concentration. [H^+^], hydrogen ion concentration. MCAv, middle cerebral artery blood velocity. $P_{CO_{2}}$, partial pressure of carbon dioxide.

In the 1990s, a collaboration between the United States National Aeronautics and Space Administration, the European Space Agency and the Deutsche Agentur fur Raumfahrtangelegenheiten (German Agency for Space Affairs) conducted a study wherein four participants were exposed to 0.007 and 0.012 $F_{I,CO_{2}}$ (∼5 and 9 mmHg $P_{I,CO_{2}}$ at 760 mmHg barometric pressure) for 24 days while comprehensive physiological measures were collected (Drummer et al., 1998; Elliott et al., 1998; Frey et al., 1998; Gundel, Drescher et al., 1998; Gundel, Parisi et al., 1998; Hoffmann et al., 1998; Manzey & Lorenz, 1998; Samel et al., 1998; Sexton et al., 1998; Sliwka et al., 1998). Sliwka et al. (1998) and Wenzel et al. (1998) measured middle (MCAv) and posterior cerebral artery blood velocities during visual stimulus (i.e., neurovascular coupling), CO_2_ rebreathing and head‐down tilt. Intracranial velocities increased by ∼35% in the first few days, returning to near normal by the seventh day in both exposures. During CO_2_ rebreathing in chronic hypercapnia, although MCAv was greater at baseline, MCAv responses (i.e., Δ MCAv) were not different from those during normocapnia. Indeed, MCAv responses to acute CO_2_, visual stimuli and head‐down tilt did not seem to be differentially affected during chronic hypercapnia. Counter to our speculation above regarding CSF compensation and the potential impacts on acute‐on‐chronic $P_{CO_{2}}$ challenges, the work by Sliwka et al. (1998) perhaps indicates that cerebral vascular reactivity is maintained despite CSF pH compensation via [HCO_3_^−^] loss. However, interpretation of these results is challenging given the limitations of transcranial Doppler (Willie et al., 2011), the methodologies used for cerebrovascular function testing, and the sampling of arterialized rather than arterial blood, or better yet CSF. Figure 6, redrawn from Sliwka et al. (1998), depicts the changes in arterialized sample acid–base balance and baseline MCAv. Thirty days of ∼3.6 mmHg $P_{I,CO_{2}}$ combined with head‐down tilt was shown to have no effect on acute CVR (Laurie et al., 2020), although the study also showed no changes in $P_{\mathrm{aC}O_{2}}$ at any time point, which probably indicates that CSF pH balance was not challenged.

### 3.2 Intracranial pressure

Given the body fluid shifts associated with spaceflight microgravity, substantial consideration has been given to the effects on cerebral perfusion pressure and ICP (Lawley et al., 2017; Marshall‐Bowman et al., 2013). In Earth gravity, acute hypercapnia can drive elevations in ICP through increased CBF and cerebral blood volume (Kim et al., 2006); however, the combination of CO_2_ and head‐down tilt does not additively elevate ICP above head‐down tilt (simulated microgravity) alone (Kurazumi et al., 2018). Moreover, it seems that CBF reactivity to CO_2_ is more related to cerebral perfusion pressure (i.e., mean arterial pressure minus ICP) than to ICP per se, at least acutely (Hauerberg et al., 2001). In rats after chronic hypercapnia (21 weeks, 0.1 $F_{I,CO_{2}}$), Kondo et al. (1999) found that the ICP response to acute hypercapnia was attenuated. This finding supports our earlier speculation that following acid–base compensation in the CSF during chronic respiratory acidosis, an acute CO_2_ challenge might result in a less severe response in the cerebrovasculature. The effects of chronic respiratory acidosis on ICP, cerebral perfusion pressure and CVR could feasibly impact the development of ocular issues, headaches and even cognition in spaceflight, and this has motivated plentiful research, albeit inconclusive (e.g., Christian et al., 2024; Laurie et al., 2017; Marshall‐Bowman et al., 2013).

Spaceflight‐associated neuro‐ocular syndrome describes a set of neurological and ocular symptoms experienced by long‐duration spaceflight astronauts (Macias et al., 2020). The precise aetiology of spaceflight‐associated neuro‐ocular syndrome remains unresolved but it is likely to result from of a number of factors, including cephalad fluid shifts, changes in ICP and other trans‐compartment pressures, issues with glymphatic drainage and hypercapnia‐induced changes in cerebral blood volume (Lee et al., 2020). Depending on the degree of CSF acid–base compensation during chronic mild respiratory acidosis, cerebral acidosis and/or elevated cerebral [HCO_3_^−^] should also be considered as potential contributors. Given the direct effect of elevated [H^+^] on cellular function and neurotransmission (Broda et al., 1959; Kraig et al., 1987; Lagadic‐Gossmann et al., 2004) even if pH is normalized, it is feasible that higher proton concentrations could still produce detrimental effects.

### 3.3 Pathological hypercapnia and cerebral blood flow

In pathological chronic respiratory acidosis, such as chronic obstructive pulmonary disease (COPD) and emphysema, reports on CBF status are mixed. Some studies find that CBF is elevated above normal (Albayrak et al., 2006; Patterson et al., 1952; Skinhøj, 1968), as would be expected with elevated $P_{\mathrm{aC}O_{2}}$ (and, presumably, elevated CSF [H^+^]), and is higher in exacerbated versus stable COPD (Yildiz et al., 2012), whereas other studies report no differences in CBF with lung disease from normative values (Cannizzaro et al., 1997) or even lower than healthy values (Jensen et al., 2002). Van de Ven et al. (2001) found higher cerebral blood volume in hypercapnic COPD compared with normocapnic COPD, yet both groups had lower cerebral blood volume than healthy control subjects. However, cerebral blood volume reactivity to CO_2_ was lowest in hypercapnic COPD versus normocapnic COPD and control subjects. The inconsistency in CBF findings among studies of pathological chronic respiratory acidosis is unavoidable given the number of possible confounding factors involved with studying such cohorts. Comorbidities, medical treatments and the direct and indirect sequelae of hypercapnia will obfuscate interpretation of the effects of long‐term respiratory acidosis alone on the cerebral vasculature.

### 3.4 Perinatal consequences of chronic CO_2_ exposure

Should mankind embark on multigenerational spaceflight endeavours, one hopes that by such a time, considerations such as ambient CO_2_ concentrations are no longer issues. The impacts of chronic CO_2_ exposure on pregnancy and development would be relevant, given that animal models of lifetime exposure to elevated CO_2_ suggest a causative role for perinatal developmental abnormalities (Haring, 1966; Sprenger & Milsom, 2021; Wyrwoll et al., 2022), bone calcium depletion (Schaefer et al., 1980), deterioration of cognitive abilities akin to hyperactivity and increased anxiousness (Burgraff et al., 2018) associated with increased corticosterone (Wyrwoll et al., 2022), increased neuronal dysfunction (Burgraff et al., 2019) and negative impacts on both structure and function of the lungs in female mice (Larcombe et al., 2021). To our knowledge, no research exists investigating the effects on CBF or cerebral vascular control following perinatal CO_2_ exposure, nor does any research exist investigating the effects of chronic CO_2_ in humans during prenatal development and across the lifetime.

## 4 FUTURE APPLICATIONS AND MITIGATION STRATEGIES

The feasibility of human spaceflight depends on a myriad of factors related to engineering and life sciences, not the least of which for human survival include radiation, micro‐/zero gravity, isolation, chronophysiological disruption and the regulation of breathable gas. With extended‐duration spaceflight and greater distances from Earth, the delivery of medical care becomes impractical. Indeed, even real‐time communication with medical ground support for directives with the occurrence of medical emergency becomes less tenable with greater distances owing to communication delays. This is part of what NASA refers to as human‐system integration architecture risk. In order to mitigate these risks, spaceflight crews will need to be trained in many specialized support systems.

As ambient pressure decreases, $P_{I,CO_{2}}$ likewise decreases. Terrestrial sea‐level barometric pressure is ∼760 mmHg, as is pressure aboard the International Space Station (NASA, 2023b), hence 0.5% CO_2_ equates to ∼3.5 mmHg $P_{I,CO_{2}}$ (e.g., Laurie et al., 2020). However, at 400 mmHg barometric pressure, the same $F_{I,CO_{2}}$ would elicit only ∼1.7 mmHg $P_{I,CO_{2}}$ (see Table 1 for $P_{I,CO_{2}}$ calculated using 400 mmHg barometric pressure), thereby widening the pressure gradient between the body and atmosphere. This would relieve the driving pressure of CO_2_ retention and attenuate the degree of acidaemia (Schaefer, 1971). This might be only a small reduction in $P_{I,CO_{2}}$, but if even low‐level CO_2_ should pose a danger across several weeks or months of exposure, this difference could meaningfully impact normal cerebral function and health. A greater fraction of inspired O_2_ would easily offset this reduction in barometric pressure to avoid hypoxia. The physiological effects of a mildly hypercapnic atmosphere could be offset by the imposition of a mild hypoxia in suits and rovers, given that a mild hypoxic ventilatory response could theoretically lower $P_{\mathrm{aC}O_{2}}$. Alternative mitigation strategies might include pharmacological activation of carbonic anhydrases (Casini et al., 2003; Supuran, 2018) or administration of bicarbonate (Adrogué & Madias, 2024) to improve acid buffering and ventilatory CO_2_ excretion; however, much more research is required to assess the long‐term safety of such interventions (Chand et al., 2021). Lessening the risk and impacts of chronically elevated CO_2_ is of the utmost importance both to achieving viable spaceflight and to its maintenance following launch. Finally, the physiological implications of chronic respiratory acidosis are relevant to many more environmental scenarios than spaceflight, such as industrial and military contexts, wildfire exposure, mining and agriculture. As such, the scope for the impact of such research is far‐reaching.

## 5 CONCLUSIONS

Transient acute increases in inspired CO_2_ represent an important physiological stressor. However, chronic exposure to inspired CO_2_ provides opportunities for respiratory and renal compensation, but depending upon the magnitude, might still lead to sustained cerebral effects. With the growing interest in government and private initiatives to colonize the Moon and Mars, the potential implications and mitigation strategies associated with chronic inspired CO_2_ exposures have become increasingly important.

## AUTHOR CONTRIBUTIONS

All authors critically reviewed the article. All authors approved the final version of this article and agree to be accountable for all aspects of the work. All persons designated as authors qualify for authorship, and all those who qualify for authorship are listed.

## CONFLICT OF INTEREST

None declared.
